# Supplementary material for: The SMC Hinge is a Selective Gate for Obstacle Bypass
Source: Nat Commun. 2025 Nov 25;16:10457. doi: 10.1038/s41467-025-65408-5 (PMC12647877; doi:10.1038/s41467-025-65408-5)
Supplement: Supplementary file 2 — Description of Additional Supplementary Files [file 41467_2025_65408_MOESM2_ESM.pdf]

Title: Supplementary Table 1

Description: List of oligonucleotides and modified DNA substrates

Title: Supplementary Table 2

Description: List of plasmids used in this study

Title: Supplementary Table 3

Description: List of bacterial strains used in this study
