# Supplementary material for: The SMC Hinge is a Selective Gate for Obstacle Bypass
Source: Nat Commun. 2025 Nov 25;16:10457. doi: 10.1038/s41467-025-65408-5 (PMC12647877; doi:10.1038/s41467-025-65408-5)
Supplement: Supplementary file 4 — Reporting Summary [file 41467_2025_65408_MOESM4_ESM.pdf]

nature portfolio

Corresponding author(s): Stephan Gruber

Last updated by author(s): Sep 17, 2025

Reporting Summary

Nature Portfolio wishes to improve the reproducibility of the work that we publish. This form provides structure for consistency and transparency in reporting. For further information on Nature Portfolio policies, see our Editorial Policies and the Editorial Policy Checklist.

Statistics

For all statistical analyses, confirm that the following items are present in the figure legend, table legend, main text, or Methods section.

n/a	Confirmed	
☐	☒	The exact sample size (n) for each experimental group/condition, given as a discrete number and unit of measurement
☐	☒	A statement on whether measurements were taken from distinct samples or whether the same sample was measured repeatedly
☒	☐	The statistical test(s) used AND whether they are one- or two-sided<br>*Only common tests should be described solely by name; describe more complex techniques in the Methods section.*
☒	☐	A description of all covariates tested
☒	☐	A description of any assumptions or corrections, such as tests of normality and adjustment for multiple comparisons
☐	☒	A full description of the statistical parameters including central tendency (e.g. means) or other basic estimates (e.g. regression coefficient) AND variation (e.g. standard deviation) or associated estimates of uncertainty (e.g. confidence intervals)
☒	☐	For null hypothesis testing, the test statistic (e.g. F, t, r) with confidence intervals, effect sizes, degrees of freedom and P value noted<br>*Give P values as exact values whenever suitable.*
☒	☐	For Bayesian analysis, information on the choice of priors and Markov chain Monte Carlo settings
☒	☐	For hierarchical and complex designs, identification of the appropriate level for tests and full reporting of outcomes
☒	☐	Estimates of effect sizes (e.g. Cohen's d, Pearson's r), indicating how they were calculated

Our web collection on statistics for biologists contains articles on many of the points above.

Software and code

Policy information about availability of computer code

Data collection	AlphaFold2 – Google CoLab; AlphaFold3 – AlphaFoldServer; VisionWorks 11.1 (Agarose gel images); Epson scan V 3.9.2.2 (Coomassie protein gels); Illumina NovaSeq 6000 (ChIP-seq data acquisition)
Data analysis	SnapGene® software V6.0.2; Fiji ImageJ 2.l.0/l.53c; Prism for Graph Pad 9.0; SeqMonk 1.48.0 (ChIP-seq data visualization); Real-time PCR miner 4.0 (ChIP-qPCR data analysis)

For manuscripts utilizing custom algorithms or software that are central to the research but not yet described in published literature, software must be made available to editors and reviewers. We strongly encourage code deposition in a community repository (e.g. GitHub). See the Nature Portfolio guidelines for submitting code & software for further information.

Data

Policy information about availability of data

All manuscripts must include a data availability statement. This statement should provide the following information, where applicable:

- Accession codes, unique identifiers, or web links for publicly available datasets
- A description of any restrictions on data availability
- For clinical datasets or third party data, please ensure that the statement adheres to our policy

Chromatin immunoprecipitation coupled to sequencing (ChIP-seq) data are deposited at the NCBI GEO database under the accession code: GSE303091
All other raw data is accessible through Mendeley: doi:10.17632/jshbprb4sh.1.

Research involving human participants, their data, or biological material

Policy information about studies with human participants or human data. See also policy information about sex, gender (identity/presentation), and sexual orientation and race, ethnicity and racism.

Reporting on sex and gender	Not applicable.
Reporting on race, ethnicity, or other socially relevant groupings	Not applicable.
Population characteristics	Not applicable.
Recruitment	Not applicable.
Ethics oversight	Not applicable.

Note that full information on the approval of the study protocol must also be provided in the manuscript.

Field-specific reporting

Please select the one below that is the best fit for your research. If you are not sure, read the appropriate sections before making your selection.

☒ Life sciences ☐ Behavioural & social sciences ☐ Ecological, evolutionary & environmental sciences

For a reference copy of the document with all sections, see nature.com/documents/nr-reporting-summary-flat.pdf

Life sciences study design

All studies must disclose on these points even when the disclosure is negative.

Sample size	Sample size for quantification (Supplementary Fig. 6B) was chosen based on experience with similar experiments and resource limitations. The statistical significance readily allows discrimination of active from inactive Wadjet.
Data exclusions	No data was excluded from the analysis.
Replication	All attempts at replication were successful. Quantitative measurements were performed in three technical replicates. Means and standard deviation are reported. All other experiments were performed at least three times from independently grown cultures or purified proteins with comparable outcomes. Two biological replicates of ChIP were sequenced.
Randomization	Experiments did not involve the allocation of groups and randomization was not relevant.
Blinding	Experiments did not involve the allocation of groups and blinding was not relevant.

Reporting for specific materials, systems and methods

We require information from authors about some types of materials, experimental systems and methods used in many studies. Here, indicate whether each material, system or method listed is relevant to your study. If you are not sure if a list item applies to your research, read the appropriate section before selecting a response.

Materials & experimental systems

n/a	Involved in the study
☐	☒ Antibodies
☐	☒ Eukaryotic cell lines
☒	☐ Palaeontology and archaeology
☒	☐ Animals and other organisms
☒	☐ Clinical data
☒	☐ Dual use research of concern
☒	☐ Plants

Methods

n/a	Involved in the study
☐	☒ ChIP-seq
☒	☐ Flow cytometry
☒	☐ MRI-based neuroimaging

Antibodies

Antibodies used: Custom-made rabbit anti-bsuScpB polyclonal serum

Validation: Specificitiy of chromatin immuno-precipitation was validated using control samples lacking the respective antigen (scpB mutants) Fig. 3C).

Eukaryotic cell lines

Policy information about cell lines and Sex and Gender in Research

Cell line source(s): Sf9 insect cell line for protein production

Authentication: No authentication was done.

Mycoplasma contamination: Cell lines were not tested for Mycoplasma contamination.

Commonly misidentified lines (See ICLAC register): None.

Plants

Seed stocks: Not applicable.

Novel plant genotypes: Not applicable.

Authentication: Not applicable.

ChIP-seq

Data deposition

☒ Confirm that both raw and final processed data have been deposited in a public database such as GEO.

☐ Confirm that you have deposited or provided access to graph files (e.g. BED files) for the called peaks.

Data access links
May remain private before publication.: https://www.ncbi.nlm.nih.gov/geo/query/acc.cgi?acc=GSE303091

Files in database submission: 60B1_WT_INP, 60B1_hinge3A_INP, 60B1_scpBdel_INP, 60B1_WT_IP, 60B1_hinge3A_IP, 60B1_scpBdel_IP,

Genome browser session (e.g. UCSC): SeqMonk

Methodology

Replicates: ChIP-qPCR was performed in biological triplicates, demonstrating high reproducibility. Two samples each were sequenced with data from a representative example shown in the figures. Data for both replicates are available at the NCBI GEO database.

Sequencing depth: 20M total reads (4M bp genome)

Antibodies	Custom-made rabbit anti-bsuScpB polyclonal serum
Peak calling parameters	Not applicable.
Data quality	Mean quality score 42.0-42.6
Software	Galaxy (https://usegalaxy.org/) bowtie2, samtools sort, SeqMonk visualizaton
